# Cancer Vaccine Therapy Using Carcinoembryonic Antigen - expressing Dendritic Cells generated from Induced Pluripotent Stem Cells

**DOI:** 10.1038/s41598-018-23120-z

**Published:** 2018-03-15

**Authors:** Junya Kitadani, Toshiyasu Ojima, Hiromitsu Iwamoto, Hirotaka Tabata, Mikihito Nakamori, Masaki Nakamura, Keiji Hayata, Masahiro Katsuda, Masayasu Miyajima, Hiroki Yamaue

**Affiliations:** 1 Second Department of Surgery, Wakayama Medical University, School of Medicine, Wakayama, 641–8510 Japan; 2 Laboratory Animal Center, Wakayama Medical University, School of Medicine, Wakayama, 641–8510 Japan

## Abstract

Clinical application of dendritic cell (DC) vaccine therapy is hindered by the need for a large quantity of DCs generated from peripheral blood monocytes of the patient. We investigated whether genetically modified human induced pluripotent stem cell (iPSC)-derived dendritic cells (hiPSDCs) expressing carcinoembryonic antigen (CEA) could induce CEA-specific cytotoxic T cells in a human model and whether genetically modified mouse iPSDCs (miPSDCs) expressing CEA showed an actual antitumor effect using a CEA transgenic mouse model. We differentiated hiPSDCs from iPSCs of three healthy donors and transduced CEA cDNA into the hiPSDCs. The surface marker expression, cytokine secretion and migratory capacity of the hiPSDCs were equivalent to those of human monocyte-derived DCs (hMoDCs). Cytotoxic T cells activated by hiPSDCs-CEA exhibited CEA-specific cytotoxic activity against the target cells expressing CEA. Furthermore, in the CEA transgenic mouse model, cytotoxic T cells activated in mice immunized with miPSDCs-CEA displayed CEA-specific cytotoxic activity against MC38-CEA. In the subcutaneous tumour model, vaccination with miPSDCs-CEA achieved a significant growth inhibitory effect on MC38-CEA. No adverse events caused by the administration of miPSDCs were observed. Genetic modification of iPSDCs, inducing the expression of CEA, is a promising tool for clinical applications of vaccine therapy for treating gastrointestinal cancer patients.

## Introduction

Dendritic cells (DCs) are the most potent antigen-presenting cells, and they play a major role in the initiation of antitumor immune responses^1^. DC activity is primarily dependent upon antigen-specific CD8^+^ T cells, which, among other functions, generate cytotoxic T cells to reject cancer. We previously demonstrated that DCs adenovirally transduced with the tumour associated antigen (TAA) gene effectively induced TAA-specific cytotoxic T cells to elicit antitumor responses *in vivo* and *in vitro*^2–5^. We suggest that this strategy is ideal for cancer immunotherapy for patients with gastrointestinal tumours.

The clinical application of DC vaccine therapy, however, is hindered by the need for a large quantity of DCs generated from the peripheral blood monocytes of the patient. Therefore, leukapheresis must be performed, which is burdensome for the patients. An additional issue is that in cancer patients, the number of DCs is reduced and the function of DCs is in decline^6,7^.

The inductions of iPSCs were reported by Yamanaka *et al*. since 2006^8–10^. To that end, we used induced pluripotent stem cells (iPSCs) to obtain DCs. Several groups have reported methods for differentiating DCs from iPSCs (iPSDCs) in mouse and human studies^11–15^. Our previous study indicated that mouse iPSDCs have a capacity for maturation and migration equal to that of bone marrow DCs. Furthermore, TAA-specific cytotoxic T cells were generated by the administration of genetically modified iPSDCs expressing TAA in a mouse melanoma model^16^. This new method may therefore be promising for future clinical applications in cancer immunotherapy. However, no studies have yet examined the induction of cytotoxic T cells against gastrointestinal tumours by vaccination with iPSDCs.

Carcinoembryonic antigen (CEA) is a heavily glycosylated oncofetal antigen overexpressed in human adenocarcinomas, particularly in gastrointestinal cancer, making it a valuable target for immunotherapy specific for gastrointestinal cancer^17–20^. Two decades ago, our laboratory performed its first vaccine study using CEA, including a phase I trial of cancer immunotherapy using CEA peptide-pulsed DCs and basic research on DC vaccine therapy with the transduced CEA gene using a CEA transgenic mouse model^3,4,21^. In the near future, we aim to develop a drug for cancer immunotherapy targeting CEA.

In this study, we investigated whether genetically modified human iPSDCs (hiPSDCs) expressing CEA could induce CEA-specific cytotoxic T cells against cancer cell lines endogenously expressing CEA in an *in vitro* model using healthy volunteers. Furthermore, we established an *in vivo* tumour model using CEA transgenic mice as a preclinical experiment. We transduced mouse iPSDCs (miPSDCs) with the CEA gene and examined whether these genetically modified DCs could induce strong therapeutic antitumor immune responses against tumour cells expressing CEA in CEA transgenic mice. Immunotherapies using iPSCs must strike a balance between desirable antitumor responses and unwanted adverse reactions because the immunogenicity of iPSCs and their malignant transformation have not been vigorously examined^22^. Therefore, we also assessed the autoimmune reactions and adverse reactions in mice immunized with miPSDCs. The purpose of this study was to assess the feasibility of this vaccination system using genetically modified iPSDCs expressing CEA.

## Results

### Human model

#### Generation of hiPSDCs from healthy human iPSCs

**Figure 1:**
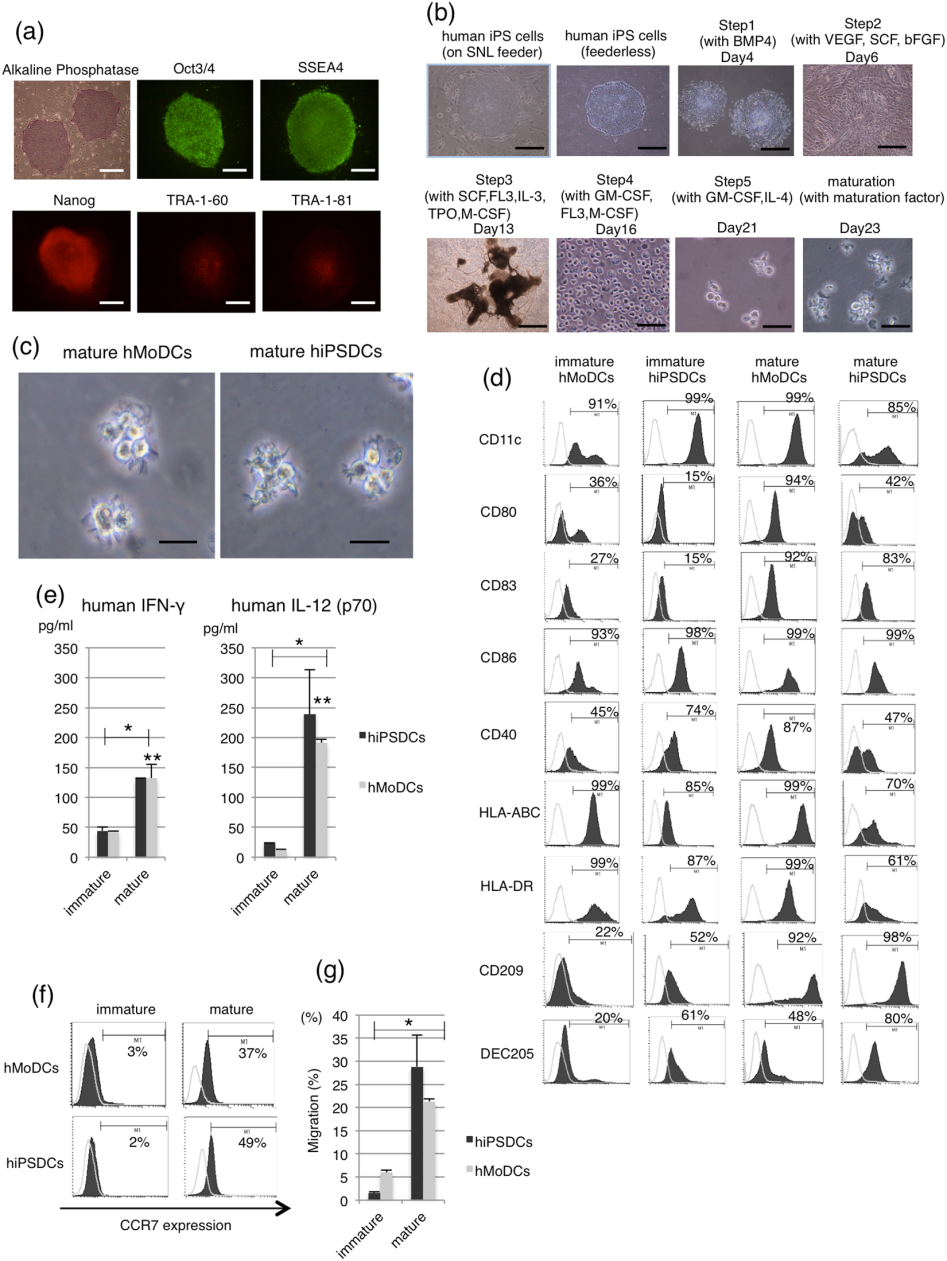
Maturation stability of hMoDCs and hiPSDCs. (**a**) Characterization of human iPSCs. Alkaline phosphatase staining and fluorescent staining with undifferentiated markers showed pluripotency of human iPSCs. Scale bars = 80 μm. (**b**) The schematic diagram of differentiation protocol for hiPSDCs. Scale bars = 80 μm (Before Day 16). Scale bars = 20 μm (After Day 21). (**c**) Morphology of mature hMoDCs on day seven and mature hiPSDCs on day 23. Scale bars = 20 μm. (**d**) Surface phenotypes of hMoDCs and hiPSDCs. Histograms show the staining results of specific antibodies (black) and isotype-matched controls (thin lines). (**e**) Secretion of human IFN-γ and human IL-12 (p70) from hMoDCs and hiPSDCs. Data represent the mean ± SD (three donors for each group). *Significantly higher than the immature DCs. (*P* < 0.01) **No significant differences between the mature hMoDCs and mature hiPSDCs (*P* > 0.05). (**f**) Migration capacity of hMoDCs and hiPSDCs, which were examined by the expression of CCR7, using flow cytometry. Histograms show staining results of specific antibodies (black) and isotype-matched controls (thin lines). (**g**) Chemotactic assay *in vitro*. Data represent the mean ± SD (three donors for each group). Almost 30% of the mature hiPSDCs migrated in response to macrophage inflammatory protein 3β chemokine in transwells, while less than 5% of the immature hiPSDCs did so. *Significantly higher than the immature DCs (*P* < 0.01). A similar migratory capacity was observed in hMoDCs and hiPSDCs.

We were able to establish undifferentiated iPSCs from the fibroblasts of three healthy donors using the Sendai virus vector, and we succeeded in inducing the differentiation of these iPSCs into hiPSDCs. Alkaline phosphatase staining and fluorescent staining with undifferentiated markers showed pluripotent status of hiPSCs induced from three healthy donors (Fig. 1a). The schematic diagram of differentiation protocol for hiPSDCs was displayed in Fig. 1b. These iPSCs were maintained on tissue culture dishes coated with growth factor-reduced Matrigel in mTeSR1 serum-free medium. The protocol consisted of five sequential steps. In step 1, primitive streak cells were induced from undifferentiated iPSCs and then differentiated into hemangioblast-like hematopoietic progenitors in step 2. After seven days, in step 3, dome-shaped structures containing CD43 positive cells were found. After three days, in step 4, the majority of the floating cells were CD14 positive monocyte-like cells. CD14 positive cells were differentiated at an average rate of 1.5 × 10^6^ cells per 100 mm culture dish. Cells with protrusions appeared in step 5 of the immature DC stage, and then, after the addition of maturation cocktails of recombinant human (rh) IL-6, rhTNF-α, rhIL-1β and prostaglandin E2 (PGE2) for 48 hours, the protrusion increased noticeably in the mature DC stage. The resulting mature hiPSDCs were morphologically similar to mature human monocyte–derived DCs (hMoDCs; Fig. 1c). Flow cytometric analysis demonstrated that the immature hiPSDCs expressed a high level of CD11c, similar to immature hMoDCs. The immature hiPSDCs expressed CD86, CD40, HLA-ABC and HLA-DR but did not express CD80 or CD83. After stimulation with maturation cocktails, hiPSDCs expressed high levels of co-stimulating molecules CD83, CD86 and major histocompatibility complex molecules HLA-ABC and HLA-DR as well as those of hMoDCs. Although mature hiPSDCs also expressed co-stimulating molecules CD80 and CD40, the expressing levels were lower than those of hMoDCs (Fig. 1d). Furthermore, flow cytometric analysis demonstrated that mature hiPSDCs expressed a high level of CD209 and DEC205, which were characteristic markers for dendritic cells, although the immature hiPSDCs expressed a low level of CD209 and DEC205. These expressions of CD209 and DEC205 were similar to those of hMoDCs. All experiments were performed using material from the three subjects to confirm the reproducibility of the results, and similar results were obtained.

#### Secretion of IFN-γ and IL-12 (p70) from DCs

We examined the secretion of hIFN-γ and hIL-12 (p70) from hMoDCs or hiPSDCs after stimulation with maturation cocktails. Both immature hMoDCs and hiPSDCs secreted low levels of hIFN-γ and hIL-12 (p70), whereas the levels secreted from mature hMoDCs and hiPSDCs were significantly higher (*P* < 0.01; Fig. 1e). There were no significant differences in secretion level between the mature hMoDCs and hiPSDCs (*P* > 0.05).

#### Migratory capacity of DCs

To confirm the migratory ability of the hMoDCs and hiPSDCs, the surface expression of CCR7 was assessed using flow cytometry. The percentage of positively stained cells was less than 10% for the immature hMoDCs and hiPSDCs, whereas the percentage for the mature hMoDCs and hiPSDCs was increased to 37% and 49%, respectively (Fig. 1f). The migratory capacity of hiPSDCs was assessed in an *in vitro* chemotactic assay. Almost 30% of the mature hiPSDCs migrated in response to the macrophage inflammatory protein (MIP) 3β chemokine in transwells, whereas less than 5% of immature hiPSDCs did so. The mature hiPSDCs exhibited significantly higher migration capacity compared with the immature hiPSDCs (*P* < 0.01). A similar trend for migratory capacity was observed in hMoDCs (Fig. 1g).

#### Expression of CEA in genetically modified DCs

**Figure 2:**
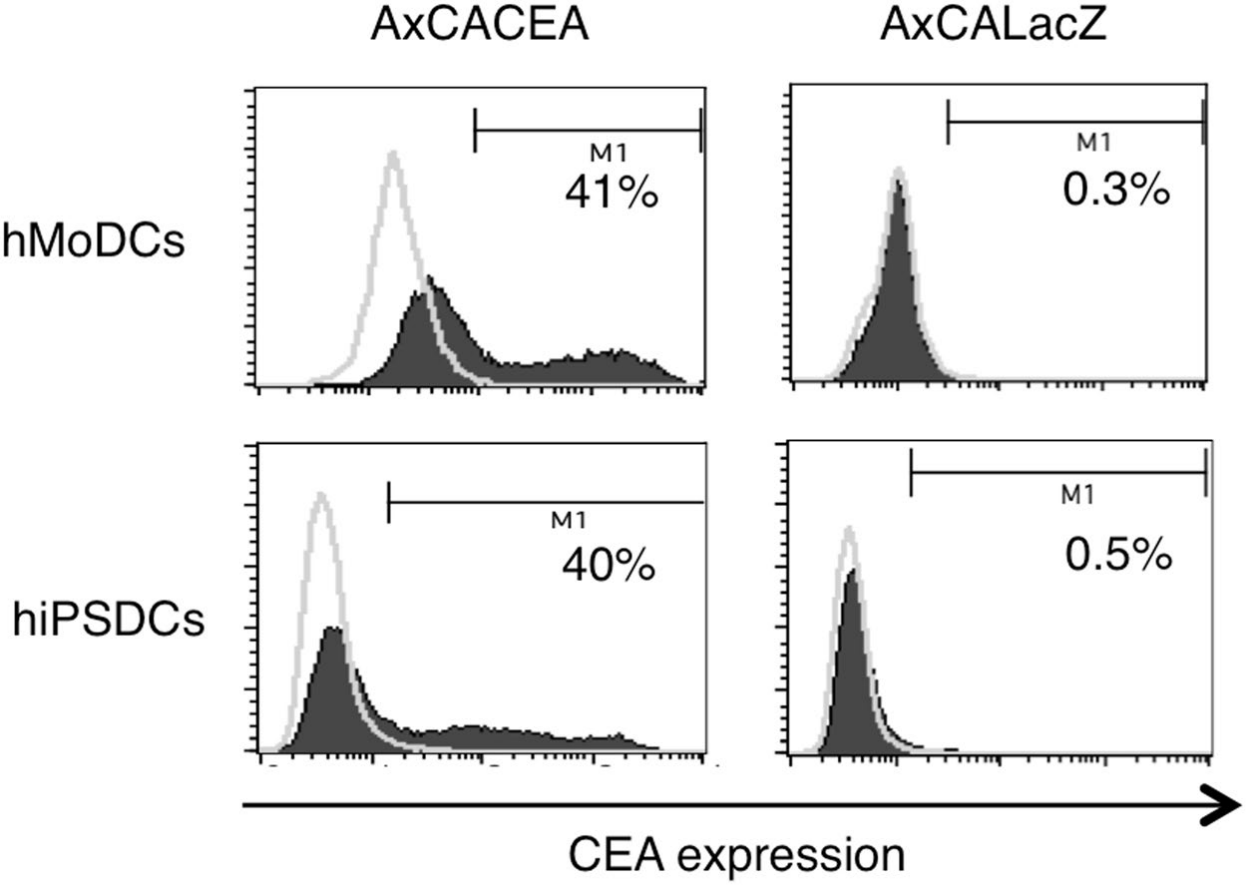
Expression of CEA in the genetically modified DCs. The intracellular expression of CEA in the genetically modified hMoDCs and hiPSDCs. DCs were analysed by intracellular staining and flow cytometry. Histograms show the staining results of CEA (black) and FITC-matched controls (thin lines).

Immature DCs were transduced with each recombinant adenoviral vector using a centrifugal method. Briefly, DCs mixed with adenoviral vectors at 100 multiplicities of infection were centrifuged at 2000 × *g* at 37 °C for 2 hours. Our previous studies showed that the appropriate multiplicities of infection for AxCACEA and AxCALacZ were 100, so we fixed the values at 100 for these vectors in this study. The resulting genetically modified DCs were analysed using intracellular CEA-staining flow cytometry to compare the transfection efficiency of the CEA gene between hMoDCs and hiPSDCs. The percentage of positively stained cells among the hMoDCs and hiPSDCs was 41% and 40%, respectively (Fig. 2).

#### Induction of CEA-specific cytotoxic T cells and cytotoxicity in healthy human donors

**Figure 3:**
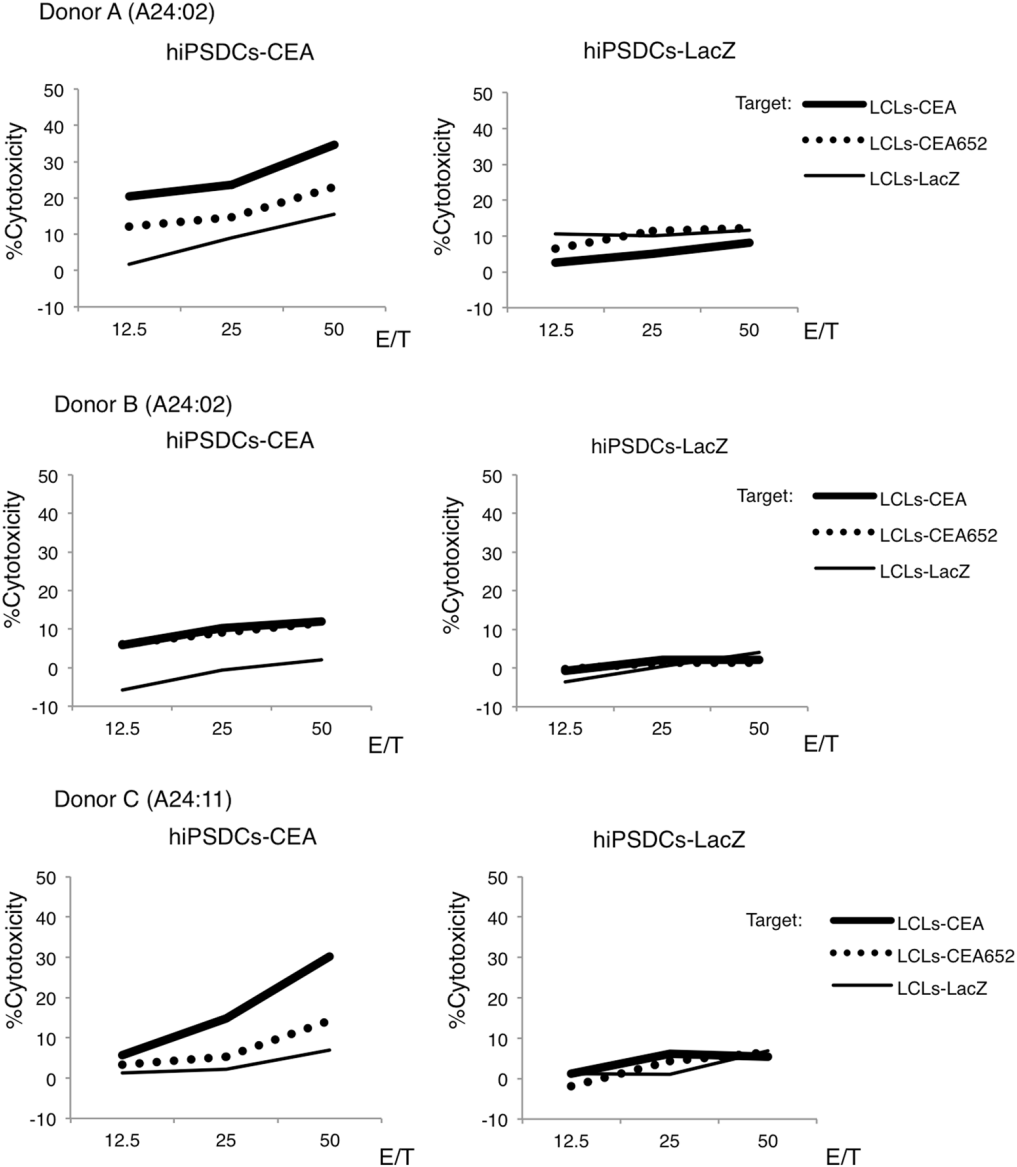
Cytotoxic activity of cytotoxic T cells generated from hiPSDCs-CEA. CD8^+^ cytotoxic T cells were sorted using an autoMACS Pro Separator after three cycles of stimulation of autologous peripheral blood mononuclear cells by genetically modified DCs, and the cytotoxic activity was tested using a 4-h ^51^Cr release assay. Cytotoxic T cells induced by hiPSDCs-CEA from each of the three HLA-A24-positive donors exhibited cytotoxic activity against LCLs-CEA and LCLs-CEA652, whereas they exhibited no cytotoxicity against LCLs-LacZ. Cytotoxic T cells induced by hiPSDCs-LacZ from each HLA-A24-positive donor exhibited no cytotoxic activity against LCLs-CEA, LCLs-CEA652, or LCLs-LacZ.

**Figure 4:**
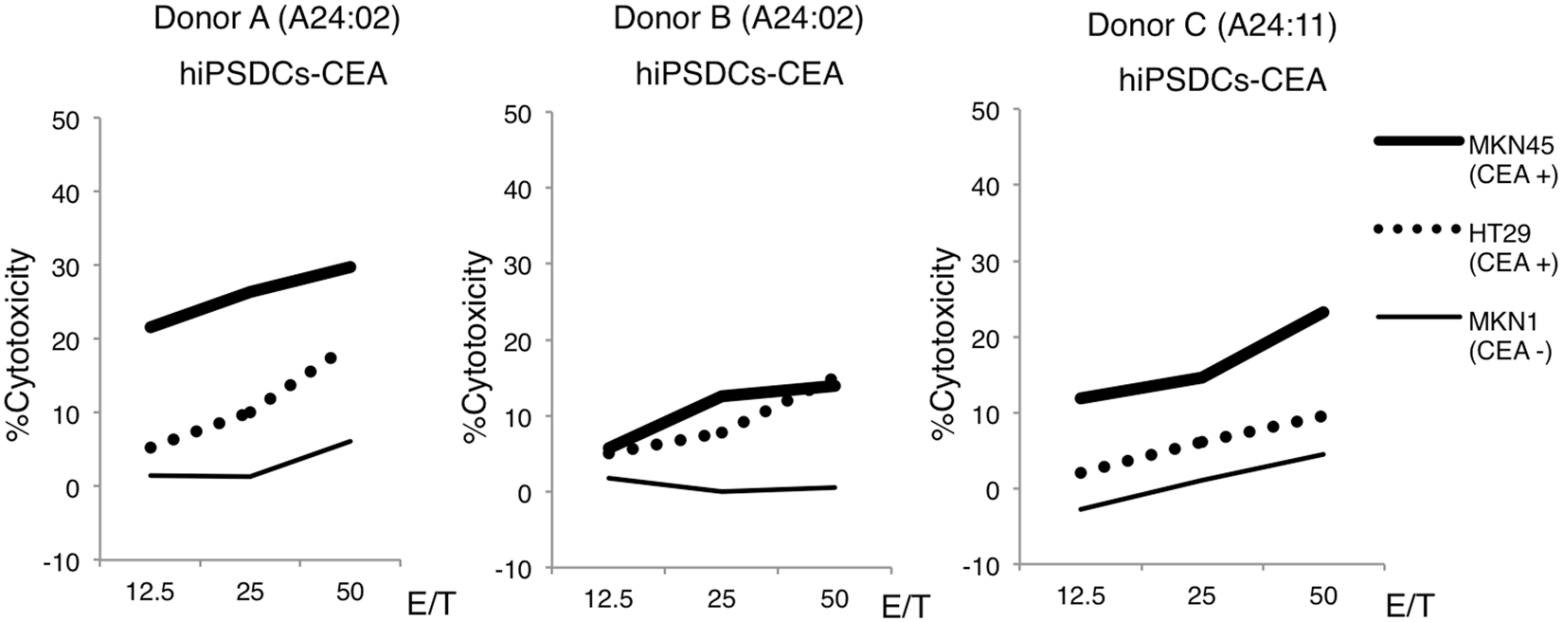
Cytotoxic activity of cytotoxic T cells generated from hiPSDCs-CEA against gastrointestinal cancer cell lines endogenously expressing CEA. The cytotoxic T cells induced by hiPSDCs-CEA from each of the three HLA-A24-positive donors exhibited cytotoxic activity against MKN45 and HT29, which endogenously expressed CEA and possessed the HLA-A24 allele, whereas they showed no cytotoxicity against MKN1, which did not endogenously express CEA and possessed the HLA-A24 allele.

The inductions of CEA-specific cytotoxic T cells were performed in auto models. Actually, hiPSDCs as effectors and T cells as responders were obtained from the same donor. Furthermore, LCLs, which were induced using EBV, were also obtained from the same donor. HLA alleles of these cells were absolutely matched. Otherwise, MKN45, HT29 and MKN1 were established cancer cell lines, which had HLA-A24 allele. After three cycles of re-stimulation of autologous peripheral blood mononuclear cells by genetically modified hiPSDCs, CD8^+^ cytotoxic T cells were sorted. The cytotoxicity of cytotoxic T cells was assessed using a ^51^Cr release assay. Experiments were performed three times from three each donor, and similar results were obtained. The highest cytotoxic activity against target cells of three times of ^51^Cr release assay for each donor was shown in Figs 3 and 4, respectively. From all three healthy donors, the cytotoxic T cells induced by hiPSDCs-CEA exhibited cytotoxic activity against lymphoblastoid cells (LCLs)-CEA and LCLs-CEA652, but they exhibited no cytotoxicity against the control, LCLs-LacZ (Fig. 3). Our results showed that CTLs induced by hiPSDCs-CEA could recognize both the CEA-specific peptide bound by HLA-A24 and multiple epitope peptides of LCLs endogenously expressing CEA gene. However, the cytotoxicities against CEA expressing target cells were weaker in donor B than the other donors. In addition, the cytotoxic T cells exhibited strong cytotoxic activity against MKN45 and HT29 cells, which endogenously express CEA and possess the HLA-A24 allele; in contrast, the cytotoxic T cells exhibited no cytotoxicity against MKN1 cells, which do not endogenously express CEA but do possess the HLA-A24 allele (Fig. 4). The cytotoxic T cells induced by hiPSDCs-LacZ did not exhibit cytotoxic activity against any of the target cells. These results suggest that the cytotoxic T cells induced by genetically modified hiPSDCs expressing CEA could exhibit HLA-restrictive and CEA-specific cytotoxic activity.

### Animal model

#### Cytotoxic activity of CD8^+^ cytotoxic T cells in CEA transgenic mice immunized with miPSDCs expressing the CEA gene

We used mouse iPS cell line iPS-MEF-Ng-20D-17, which was derived from C57/BL6 MEFs. This study using CEA transgenic mice (C57BL/6J-TgN [CEA Ge] 18FJP) were performed in syngeneic mouse model in the context of H2K^b^. Our previous study demonstrated the procedure for inducing the differentiation of miPSCs (iPS- MEF-Ng-20D-17) into mature miPSDCs using OP9 feeder cells and indicated that miPSDCs have a capacity for maturation, secretion of cytokine, migration and antigen presenting ability on equal to that of mouse bone marrow DCs^16^.

**Figure 5:**
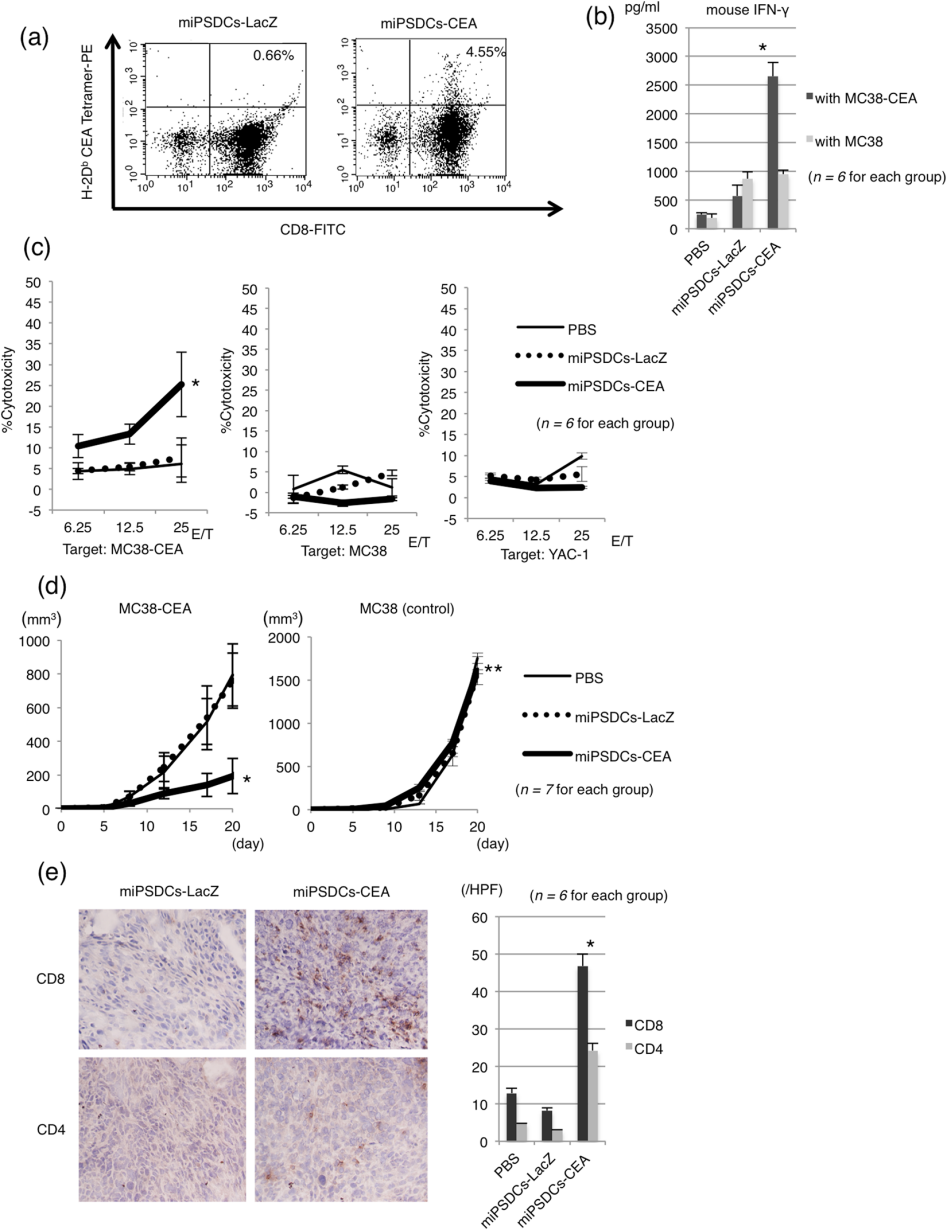
Cytotoxic activity of cytotoxic T cells induced by miPSDCs expressing CEA and therapeutic efficacy in CEA transgenic mice. (**a**) Splenocytes were removed from mice administered with genetically modified DCs and cultured with MC38-CEA added to rmIL-2. After three days, the culture cells were stained with tetramers of H-2D^b^-CEA-EAQNTTYL in combination with anti-CD8 mAb and PerCP-Cy5.5-conjugated anti-CD3 mAb, and analysed using flow cytometry. (**b**) The specific quantification of IFN-γ secretion in the presence of CEA-expressing target cells. On day 14, pools of splenocytes from CEA transgenic mice were cultured with control MC38 or MC38-CEA for 48 hours. Splenocytes were cultured with an effector-to-target ratio of 20:1, and IFN-γ secretion was measured by ELISA. *Splenocytes immunized with miPSDCs-CEA secreted significantly more IFN-γ in the presence of MC38-CEA than MC38 (*P* < 0.001; *n* = 6 for each group). (**c**) The cytotoxicity of CD8^+^ cytotoxic T cells in the CEA transgenic mice administered with genetically modified DCs (PBS, thin line; miPSDCs-LacZ, dotted line; miPSDCs-CEA, bold line). A ^51^Cr release assay was performed to assess cytotoxicity against MC38-CEA cells. Data represent the mean ± SD (*n* = 6 for each group). *Significantly higher cytotoxicity against MC38-CEA in the mice administered with genetically modified miPSDCs-CEA than those immunized with PBS and miPSDCs-LacZ (*P* < 0.01). (**d**) Tumour growth inhibitory effect in mice administered with genetically modified miPSDCs in the subcutaneous tumour model (*n* = 7 for each group). Genetically modified miPSDCs are follows: PBS (thin line), miPSDCs-LacZ (dotted line), miPSDCs-CEA (bold line). Data represent the mean ± SD of mice that developed tumours in each group. *Significantly higher therapeutic efficacy than that observed in mice immunized with PBS and miPSDCs-LacZ (*P* < 0.01). **Vaccination with genetically modified iPSDCs expressing CEA showed no therapeutic efficacy against MC38 (day 20, *P* > 0.05). (**e**) Infiltration of both CD8^+^ and CD4^+^ T cells into the subcutaneous tumour tissues. Tumour implantations and vaccinations of genetically modified miPSDCs were performed as the schedule for the tumour challenges. Seven days after vaccination of genetically modified miPSDCs, subcutaneous tumours were removed and immunostained with anti-CD4 or anti-CD8 mAb. *In mice treated with miPSDCs-CEA, both CD8^+^ and CD4^+^ T cell infiltration into subcutaneous tumour tissues was significantly more dense compared with mice treated with miPSDCs-LacZ (*P* < 0.01; *n* = 6 for each group).

To assess the possibility of DCs inducing CEA-specific cytotoxic T cells *in vivo*, a tetramer assay of the CD8^+^ T cells in the cultured splenocytes isolated from immunized mice was performed. The results showed that 0.66% and 4.55% of the CD8^+^ T cells in the cultured splenocytes isolated from mice injected with miPSDCs-LacZ and miPSDCs-CEA, respectively, were positively stained with the tetramers of H-2D^b^-CEA-EAQNTTYL (Fig. 5a). Next, we cultured splenocytes from immunized mice either in the presence of control MC38 cells or the target MC38-CEA cells for 48 hours and then measured the mouse IFN-γ secretion by ELISA. Splenocytes immunized with miPSDCs-CEA secreted significantly higher IFN-γ in the presence of MC38-CEA than MC38 (*P* < 0.001; Fig. 5b). A ^51^Cr release assay was performed to assess the cytotoxicity against MC38-CEA cells by CD8^+^ cytotoxic T cells in the splenocytes of CEA transgenic mice (*n* = 6 for each group) immunized with genetically modified miPSDCs or PBS. The CD8^+^ cytotoxic T cells in mice immunized with miPSDCs-CEA expressed significantly higher cytotoxic activity against the MC-38 CEA cells than those in mice immunized with miPSDCs-LacZ or PBS (E/T: 25, *P* < 0.01). On the other hand, the CD8^+^ cytotoxic T cells in mice administered with genetically modified miPSDCs and PBS exhibited no cytotoxicity against MC38 or YAC-1 (NK-sensitive target) (Fig. 5c).

#### Therapeutic effect of miPSDCs expressing CEA in the subcutaneous tumour model

We used treatment schedules in the subcutaneous tumour model to assess the therapeutic efficacy of administrating genetically modified miPSDCs or PBS against MC38-CEA cells (*n* = 7 for each group). The administration of miPSDCs-CEA showed significantly higher tumour inhibitory efficacy compared with the other controls (day 20, *P* < 0.01; Fig. 5d). In contrast, vaccination with genetically modified miPSDCs expressing CEA showed no therapeutic efficacy against MC38 (day 20, *P* > 0.05).

#### Histological analysis of tumour tissue in mice immunized with genetically modified miPSDCs

In a histological analysis of the tumour specimens, we observed significantly more infiltration of CD8^+^ and CD4^+^ T cells in the tumour tissues of mice immunized with miPSDCs-CEA than those of mice immunized with miPSDCs-LacZ or PBS, based on the average of 10 high-power fields (*n* = 6 for each group, *P* < 0.01; Fig. 5e). In addition, there were no significant differences between the mice immunized with miPSDCs-LacZ and PBS. This result may suggest that both CD8^+^ and CD4^+^ T cells were involved because of the antitumor effect induced by miPSDCs-CEA against MC38-CEA (Fig. 5e).

#### Evaluation of adverse events in mice immunized with genetically modified miPSDCs

After the administration of genetically modified miPSDCs, all mice remained healthy with no loss of body weight. In the mice administered with genetically modified miPSDCs, symptoms such as enteritis and diarrhoea were not observed. Novel cancer due to the vaccination of genetically modified miPSDCs was not observed in any of the mice, which were followed up for 40 days after the administration. Additionally, it was histologically confirmed that there was no inflammatory change in the large intestinal mucosa of CEA transgenic mice treated with genetically modified miPSDCs vaccine (Table 1).

**Table 1:** Evaluation of adverse events in mice immunized with genetically modified miPSDCs.

	PBS (n = 7)	miPSDCs-LacZ (n = 7)	miPSDCs-CEA (n = 7)
diarrhoea	0	0	0
vomiting	0	0	0
anorexia	0	0	0
body weight loss	0	0	0
teratoma formation	0	0	0
autoimmune colitis	0	0	0
(activity index for inflammation)	0	0	0
**Inflammatory activity**	**Score**	**Histopathology defining characteristics**	
Inactive/quiescent/normal	0	No epithelial infiltration by neutrophils	
Mildly active	1	Neutrophil infiltration of <50% of sampled crypts or cross sections, no ulcers or erosions	
Moderately active	2	Neutrophil infiltration of ≧50% of sampled crypts or cross sections, no ulcers or erosions	
Severely active	3	Erosion or ulceration, irrespective of other features	

## Discussion

In this study using healthy volunteers, cytotoxic T cells induced by *in vitro* stimulation with hiPSDCs transduced with the full-length CEA gene exhibited CEA-specific cytotoxicity against not only autologous LCLs but also HLA-restrictive gastrointestinal cancer cell lines naturally expressing CEA.

Currently, typical DC vaccine therapy requires 1 to 5 × 10^7^ DCs to be administered at a time^23–25^. In our differentiation method, approximately 1 × 10^7^ DCs can be prepared per dish, so it is easy to obtain enough DCs for a single administration. In addition, in functional terms, the hiPSDCs were matured with maturation cocktails of rhIL-6, rhTNFα, rhIL-1β and PGE2. Mature hiPSDCs expressed co-stimulating molecules or major histocompatibility complex molecules secreted adequate amounts of IFN-γ and IL-12 (p70), similar to hMoDCs. Although we measured type I IFNs levels of hiPSDCs, neither immature nor mature hiPSDCs almost produced type I IFNs. These results showed that hiPSDCs were similar to conventional DCs rather than plasmacytoid DCs mainly producing type I IFNs. Furthermore, the hiPSDCs exhibited a capacity for migration similar to that of hMoDCs. Because the maturation factors influence the maturation capacity of DCs, other representative maturation factors (i.e., rhTNFα, lipopolysaccharide, OK432 or polyinosinic-polycytidylic acid) were also examined. In our experiments, the expression of Toll-like receptors 2, 3 and 4 was observed in hiPSDCs, and constant maturation occurred by the addition of these cytokines. In particular, in hiPSDCs matured with cytokine cocktails, the expression of CD83 and CCR7 and the secretion of IL-12 (p70) were higher than in hiPSDCs matured with other maturation factors, as previously reported in hMoDCs (Supplementary Fig. 1a,b)^26–28^. Based upon our preliminary data, we used the cytokine cocktails with rhIL-6, rhTNFα, rhIL-1β and PGE2 as optimal stimuli of hiPSDCs.

In our tests using autologous LCLs, the genetically modified hiPSDCs-CEA could induce CEA-specific cytotoxic T cells *in vitro*. In addition, the cytotoxic T cells induced by hiPSDCs-CEA were able to recognize the CEA-specific peptide CEA652, which is bound by HLA-A24. However, the cytotoxicities against CEA expressing target cells were weaker in donor B than the other donors. We consider that the selective cytotoxicity in donor B was basically within a donor’s individual difference. Furthermore, the cytotoxic T cells induced by hiPSDCs-CEA exhibited strong cytotoxic activity against HLA-A24-restricted cancer cell lines that spontaneously express CEA. Thus, the cytotoxic T cells induced by the stimulation of hiPSDCs-CEA may be able to kill HLA-A24-positive tumour cells that endogenously express CEA. For the first time, we demonstrated the inducibility of TAA-specific cytotoxic T cells by the *in vitro* stimulation of human iPSDCs expressing TAA. Therefore, human iPSDCs were demonstrated to exhibit an antigen presenting function similar to that of hMoDCs.

We subsequently established an *in vivo* subcutaneous tumour model using CEA transgenic mice to investigate the actual vaccine effect of miPSDCs adenovirally expressing CEA. Our findings indicate that vaccination with miPSDCs expressing CEA can elicit a strong therapeutic immunity. The differentiation of miPSDCs was performed by a four-step protocol using OP9 feeder cells^12,16^. In the CEA transgenic mouse model, *in vivo*–primed cytotoxic T cells induced by miPSDCs-CEA exhibited high cytotoxic activity against MC38-CEA cells but not against MC38. The non-specific killing of NK cells in these vaccine models was excluded because no cytotoxic activity was exhibited against YAC-1. Furthermore, in the subcutaneous tumour model, vaccination with miPSDCs-CEA had a significant growth inhibitory effect against MC38-CEA cells. However, these vaccinations had no antitumor effect against wild-type MC38 cells at all, indicating that vaccination with miPSDCs-CEA could induce CEA-specific antitumor immunity. In addition, we observed more intense infiltration of CD8^+^ and CD4^+^ T cells into the tumour tissues of mice immunized with miPSDCs-CEA. This finding suggests that both CD8^+^ and CD4^+^ T cells induced by miPSDCs-CEA contributed to the tumour growth inhibition, as previously reported^29,30^. We consider that CD8^+^ CTLs are necessary for tumor regression and CD4^+^ helper T cell responses play a pivotal role in activating these CD8^+^ CTLs in this vaccine models using miPSDCs expressing CEA^3,31^. However, in the future, we will need to assess whether CD4^+^and CD8^+^ T cells contribute the cytotoxicity and therapeutic effect in this miPSDCs vaccine models using the depletion assay of these populations. This was the first study to show an antitumor effect on a gastrointestinal tumour by vaccination with miPSDCs. In terms of the inducibility of CEA-specific cytotoxic T cells by *in vitro* stimulation with hiPSDCs-CEA and the antitumor effect in *in vivo* mouse models, the clinical application of this vaccination strategy using hiPSDCs-CEA may show a strong antitumor effect.

Neither weight loss nor diarrhoea was observed in the CEA transgenic mice who received the miPSDC vaccine therapy. No formation of teratomas due to administration of iPSDCs was observed either. Considering the clinical application of this strategy, it is important to confirm the lack of adverse effects. This study provides evidence based on a clinically relevant model that cytotoxic T cells induced by miPSDCs-CEA were effective against MC38-CEA cell lines and that this application is feasible without causing autoimmune colitis. One reason why there were no obvious side effects may be that CEA was overexpressed in the tumour cells compared to normal colon tissue^32^. Furthermore, no novel cancer was observed in any of the mice who were vaccinated with genetically modified miPSDCs. Since undifferentiated iPSCs strongly adhere to coated dishes, this method, in which floating cells are recovered and reseeded in new dishes, is considered to decrease the chance of including undifferentiated iPSCs^12,16^. Our autologous model may be safer and have fewer side effects than an allogenic model.

In conclusion, the cytotoxic T cells induced by human and mouse iPSDCs transduced with full-length CEA cDNA could have strong cytotoxic activity against CEA-positive target cells in human healthy donors and the CEA transgenic mouse model. A previous clinical trial using DCs found the clinical effects of this vaccine therapy to be insufficient^21^, possibly because the function of the naïve DCs used for the vaccine were in an exhausted condition. Our strategy, using iPSDCs, may overcome these weaknesses with regard to sufficient numbers and the good condition of the vaccine components. Vaccine therapy using genetically modified iPSDCs expressing CEA is a promising clinical application for the treatment of gastrointestinal cancer.

## Methods

### Human cell lines

The human gastric cancer cell lines MKN1 (HLA-A24/26) and MKN45 (HLA-A24/24) (Takara, Shiga, Japan) were cultured in RPMI1640 medium supplemented with 10% FBS and 2 mM L-glutamine. The human colon cancer cell line HT29 (HLA-A24/24) (Shionogi Pharmaceutical Co., Osaka, Japan) was cultured in McCoy’s 5a medium supplemented with 10% FBS. Autologous LCLs were generated from healthy donor peripheral blood mononuclear cells transformed by the Epstein-Barr virus, as described previously^4,5,33^.

### Generation of hiPSDCs and hMoDCs

This study was conducted according to a protocol reviewed and approved by the Ethical Committee on Human Research at Wakayama Medical University Hospital (approval no. 1594). Three healthy donors provided written informed consent before participating. Dermal fibroblasts were obtained from three healthy donors positive for HLA-A*2402, HLA-A*2402, and HLA-A*2411 to generate iPSCs. The fibroblasts were infected using Sendai virus vectors kits (CytoTune-iPS 2.0, DNAVEC Corp., Ibaraki, Japan) according to the manufacturer’s instructions^34,35^. We confirmed undifferentiated and pluripotent status of iPSCs established from three healthy donors by evaluating using alkaline phosphatase staining and fluorescent staining with undifferentiated markers (FITC anti-human Oct3/4, FITC anti-human SSEA4, PE anti-human Nanog, PE anti-Human TRA-1–60, PE anti-Human TRA-1–81). The differentiation protocol of the hiPSDCs was modified from a previously established protocol under feeder-free culture^15^. In brief, in step 1, undifferentiated iPSCs were disseminated onto a 100-mm culture dish coated with growth factor-reduced Matrigel in mTeSR1 medium supplemented with 80 ng/mL rhBMP4. In step 2, on day 4, mTeSR1 medium was replaced with StemPro-34 serum-free medium containing 2 mM L-glutamine supplemented with 80 ng/mL rhVEGF, 25 ng/mL basic FGF, and 100 ng/mL rhSCF. In step 3, on day 6, the cytokines in StemPro-34 were changed to cytokines mixed with 50 ng/mL rhSCF, 50 ng/mL rhIL-3, 5 ng/mL thrombopoietin, 50 ng/mL rhM-CSF, and 50 ng/mL rhFlt-3 ligand. In step 4, on day 13, the cytokines in StemPro-34 were changed to cytokines mixed with 50 ng/mL rhM-CSF, 25 ng/mL rhGM-CSF, and 50 ng/mL rhFlt-3 ligand. CD14 positive monocytic lineage cells were sorted using an autoMACS Pro Separator with CD14 MicroBeads on days 16 to 28. In step 5, 1.5 × 10^6^ monocytic cells/well in six-well Ultra-Low Attachment Surface plates were cultured in the StemPro-34 medium containing 25 ng/mL rhGM-CSF and 40 ng/mL rhIL-4 for 5 days. MoDCs were generated *in vitro* from the peripheral blood of healthy volunteers, as described previously^4,5^.

### Recombinant adenoviral vectors and gene transfer into DCs

The recombinant adenoviral vectors expressing CEA (AxCACEA) and LacZ (AxCALacZ) were generated by the COS-TPC method^3,4^. Immature DCs were transduced with each recombinant adenoviral vector using a centrifugal method. The genetically modified DCs transduced with adenoviral vectors were placed at 1.5 × 10^6^ cells/well in six-well Ultra-Low Attachment Surface plates in the presence of 100 ng/mL rhIL-6, 10 ng/mL rhTNFα, 10 ng/mL rhIL-1β, and 1 μg/mL PGE2 to induce final maturation for 48 hours.

### Flow cytometric analysis

The following monoclonal antibodies were used for staining: PE-conjugated anti-human CD11c, FITC-conjugated anti-human CD80, FITC-conjugated anti-human CD83, FITC-conjugated anti-human CD86, PE-conjugated anti-human CD40, PE-conjugated anti-human HLA class I molecules, PE-conjugated anti-human HLA class II molecules, PE-conjugated anti-human CD209, FITC-conjugated anti-human DEC205 and FITC-conjugated anti-human CCR7 (all from Becton Dickinson). Intracellular staining with an anti-human CEA monoclonal antibody (clone 12–140–10, Leica Biosystems, Newcastle, UK) and an FITC-conjugated anti-mouse IgG polyclonal antibody (Vector Labs, Burlingame, CA, USA) was performed using a Fixation and Permeabilization Solution Kit.

### Assays for cytokine secretion

The hiPSDCs and hMoDCs were adjusted to a concentration of 1.0 × 10^5^ cells/well and cultured on a 48-well plate for 48 hours in AIM-V medium (1 mL/well). The supernatants were then harvested, and the human IFN-γ and human IL-12 (p70) levels were measured using an IFN-γ ELISA kit and an IL-12 (p70) ELISA kit, respectively (Thermo Fisher Scientific).

### *In vitro* migration assay

We used 24-well transwell plates with 8.0-μm pore inserts to evaluate the migration capacity of hiPSDCs and hMoDCs. First, 0.6 mL of AIM-V with 100 ng/mL of MIP-3β was added to the lower chamber. MIP-3β is a small cytokine belonging to the CC chemokine family also known as chemokine ligand 19 (CCL19)^36^. Immature and mature hiPSDCs or hMoDCs were harvested and washed twice. The cells were diluted in AIM-V medium to 1.5 × 10^6^ cells/mL, and the 0.1 mL was added to the top chamber. The transwell plate was incubated for 2 hours at 37 °C and 5% CO_2_. The number of cells that migrated to the lower chamber was determined using an automated cell counter.

### Induction of CEA-specific cytotoxic T cells and cytotoxicity in healthy human donors

Autologous peripheral blood mononuclear cells were used as responder cells. On day 0, a total of 4 × 10^6^ responder cells and 2 × 10^5^ hiPSDCs-CEA or hiPSDCs-LacZ cells were mixed in AIM-V medium containing 10 ng/mL rhIL-7 and cultured in a 24-well plate at a total volume of 1 mL/well. On day 2, AIM-V medium containing 20 U/mL of rhIL-2 was added at a total volume of 2 mL/well. On days 7 and 14, the cultures were re-stimulated with gene-transduced DCs at a ratio of 20:1. After three cycles of re-stimulation, CD8^+^ cytotoxic T cells were sorted from the stimulated peripheral blood mononuclear cells on day 21 using an autoMACS Pro Separator. The cytotoxic activity of CD8^+^ cytotoxic T cells was tested using a 4-h ^51^Cr release assay. Experiments were performed three times from three each donor to confirm the reproducibility of the results as described previously^4,5^. CEA cDNA-transduced LCLs (LCLs-CEA), LacZ cDNA-transduced LCLs (LCLs-LacZ), and HLA-A24 positive human cancer cell lines (MKN45, HT29, and MKN1) were also used as target cells. CEA expression was found in MKN45 and HT29 cells, but no expression was detected in MKN1 cells^4^. The 9-mer peptide CEA652 (TYACFVSNL), derived from CEA, was purchased from Proimmune Ltd. (Oxford, UK). The CEA652 peptide was identified as HLA2402-restricted epitope peptide. CEA652 pulsed LCLs (LCLs-CEA652) were also used as a model target in order to investigate whether CTLs induced by hiPSDCs-CEA could recognize the CEA-specific peptide bound by HLA-A24.

### Animal model

#### CEA transgenic mice and mouse tumour cell lines

Six- to 8-week-old CEA transgenic mice C57BL/6J-TgN (CEA Ge) 18FJP were used for the experiments. The mouse iPS cell line iPS-MEF-Ng-20D-17 (RIKEN BioResource Center, Ibaraki, Japan) was maintained on SNL76/7 feeder cells, as described previously^16^. The mouse chemically induced colon cancer cell line MC38 transfected with human CEA (referred to as MC38-CEA and kindly provided by Dr. F. James Primus, Vanderbilt University Medical Center) was maintained in DMEM supplemented with 10% FBS and 2 ml L-glutamine^32^. We carried out all animal experiments in compliance with the Japanese Government’s Animal Protection and Management Law (no. 105) and Standards Relating to the Care and Management of Laboratory Animals and Relief of Pain (no. 88), and similarly with the guidelines for animal experiments of Wakayama Medical University. The Committee of Animal Experiments (no. 647) and the Committee of Gene Recombination (no. 27–4) of Wakayama Medical University approved this study.

#### Induction of CEA-specific cytotoxic T cells in CEA transgenic mice and CEA tetramer assay

The generation of miPSDCs was performed according to a four-step protocol using OP9 feeder cells^16^. Collected immature miPSDCs were mixed with adenovirus vectors and centrifuged at 2000 × *g* at 37 °C for 2 hours to generate genetically modified miPSDCs. CEA transgenic mice were administered once with a subcutaneous injection in the right flank containing genetically modified miPSDCs (1.0 × 10^6^ cells) suspended in 200 μL of PBS (*n* = 6 for each group). On day 14, after the spleens were removed, some of the *in vivo*–primed splenocytes (8.0 × 10^6^ cells/well) were pooled and cultured in six-well plates. The splenocytes were cocultured with MC38-CEA and 50 U/mL rmIL-2. After 3 days, the cultured cells were collected, stained with the PE-conjugated tetramers of H-2D^b^-CEA-EAQNTTYL (Medical & Biological Laboratories Co., Ltd., Nagoya, Japan) in combination with FITC-conjugated anti-CD8 mAb (Becton Dickinson) and PerCP-Cy5.5-conjugated anti-CD3 mAb (Becton Dickinson), and analysed using flow cytometry.

#### Mouse IFN-γ secretion assay

After immunization with genetically modified miPSDCs (*n* = 6 for each group), the specific quantification of mouse IFN-γ secretion in the presence of CEA-expressing target cells was performed. The spleens were removed on day 14, and pools of splenocytes from CEA transgenic mice were cultured with control MC38 or MC38-CEA for 48 hours with an effector-to-target ratio of 20:1. The secretion of mouse IFN-γ was measured by ELISA.

#### Cytotoxic activity of CD8^+^ cytotoxic T cells

Spleen cells were isolated 14 days after administration, and then the *in vivo–*primed splenocytes were pooled and cocultured with MC38-CEA, as described above (*n* = 6 for each group). After 3 days, CD8^+^ cytotoxic T cells were sorted from the splenocytes using an autoMACS Pro Separator. The cytotoxic activity of the CD8^+^ cytotoxic T cells was assessed using a 4-h ^51^Cr release assay. MC38, MC38-CEA, or YAC-1 were used as the target cells. We used YAC-1 as a negative control to experiment the non-specific cytotoxicity of NK cells in these vaccine models with genetically modified miPSDCs.

#### Tumour challenge for the subcutaneous tumour model

To assess the therapeutic efficacy of genetically modified miPSDCs, we used a pre-existing subcutaneous tumour model^32^. MC38-CEA or MC38 cells (5 × 10^5^ cells/mouse) were inoculated subcutaneously into the shaved right flank region on day 0. Genetically modified miPSDCs-CEA were injected subcutaneously into CEA transgenic mice (1.0 × 10^6^ cells/mouse; *n* = 7 for each group) on day 5. The tumour size was measured every 3 or 4 days using the following formula: (short diameter)^2^ × long diameter × 0.52. Furthermore, to explore whether an immune response was detected in the tumour microenvironment, immunohistochemical assays were performed.

Additionally, large intestinal tissues from CEA transgenic mice treated with genetically modified miPSDCs were stained with Hematoxylin-Eosin and recorded for inflammatory infiltrates from the autoimmune response. Sections were evaluated by an independent expert in conformity with the histological activity index for inflammation^20^.

### Statistical analysis

All statistical analyses were performed with the SPSS software ver. 22.0 (SPSS, Chicago, IL, USA). Quantitative results are expressed as the mean ± SD. The two-tailed Student’s *t*-test was used to determine the statistical significance of the differences, and a *P*-value < 0.05 was considered to be significant.

## Electronic supplementary material

Supplementary Figure
