# Supplementary material for: Cancer Vaccine Therapy Using Carcinoembryonic Antigen - expressing Dendritic Cells generated from Induced Pluripotent Stem Cells
Source: Sci Rep. 2018 Mar 15;8:4569. doi: 10.1038/s41598-018-23120-z (PMC5854614; doi:10.1038/s41598-018-23120-z)
Supplement: Supplementary file 1 — Supplementary Figure [file 41598_2018_23120_MOESM1_ESM.pdf]

Scientific Reports Research Articles

Cancer Vaccine Therapy Using Carcinoembryonic Antigen – expressing Dendritic Cells Generated from Induced Pluripotent Stem Cells

Junya Kitadani[1], Toshiyasu Ojima[1], Hiromitsu Iwamoto[1], Hirotaka Tabata[1], Mikihito Nakamori[1], Masaki Nakamura[1], Keiji Hayata[1], Masahiro Katsuda[1], Masayasu Miyajima[2] and Hiroki Yamaue[1]

[1] Second Department of Surgery, Wakayama Medical University, School of Medicine, Wakayama 641-8510, Japan
[2] Laboratory Animal Center, Wakayama Medical University, School of Medicine, Wakayama 641-8510, Japan

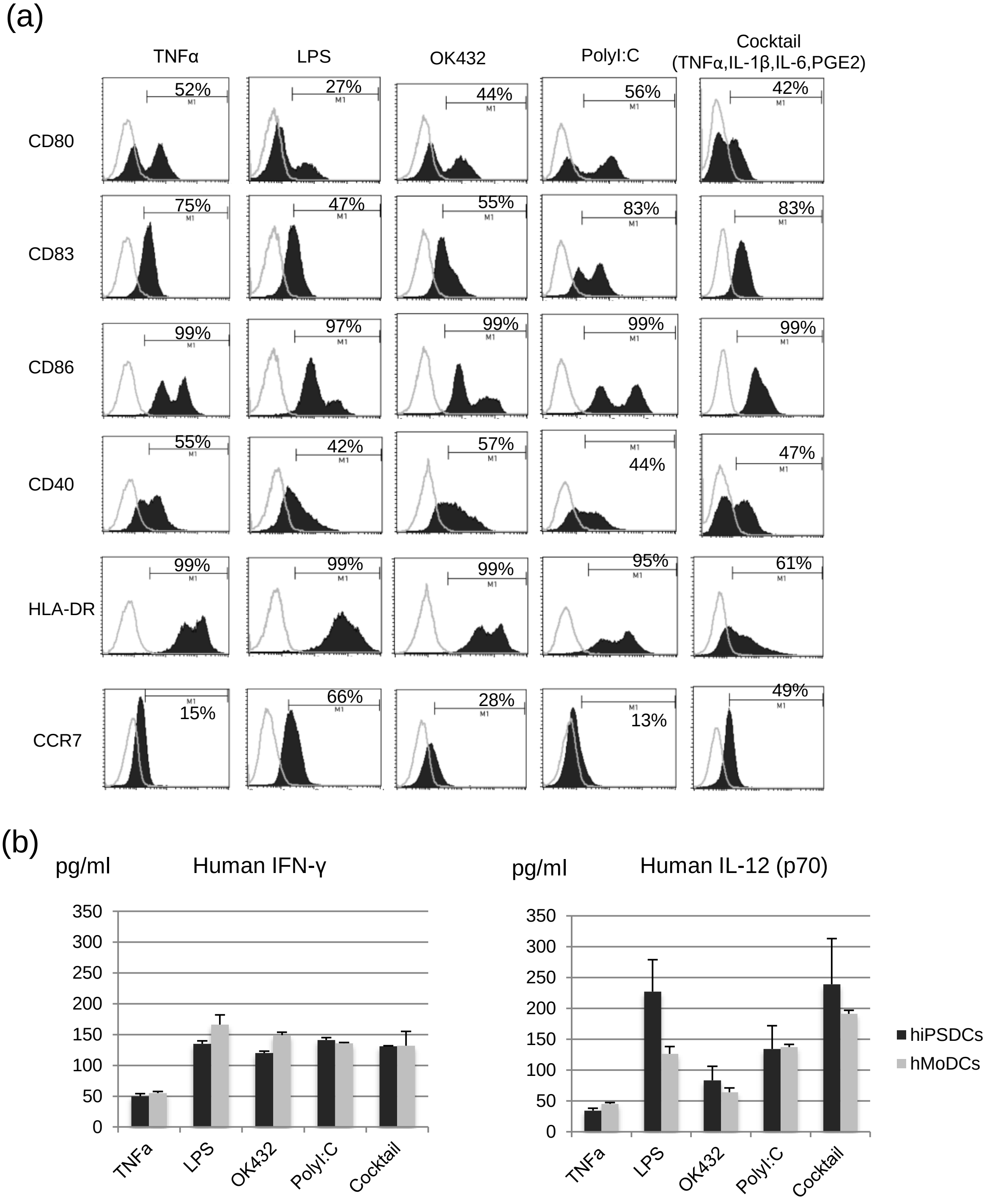

Maturation stability of hiPSDCs by other maturation factors. (a) Surface phenotypes of hiPSDCs matured by other representative maturation factors (rhTNFα, lipopolysaccharide [LPS], OK432, polyinosinic-polycytidylic acid [PolyI:C]). Histograms show the staining patterns of specific antibodies (black) and isotype-matched controls (thin lines). (b) Secretion of IFN-γ and IL-12 (p70) from hiPSDCs matured by other representative maturation factors (rhTNFα, LPS, OK432, PolyI:C). Data are mean±SD (three donors for each group).
